# Variation in the timing of Covid-19 communication across universities in the UK

**DOI:** 10.1371/journal.pone.0246391

**Published:** 2021-02-16

**Authors:** Alejandro Quiroz Flores, Farhana Liza, Husam Quteineh, Barbara Czarnecka

**Affiliations:** 1 Business and Local Government Data Research Centre, University of Essex, Colchester, Essex, United Kingdom; 2 Department of Government, University of Essex, Colchester, Essex, United Kingdom; 3 Institute for Analytics and Data Science, University of Essex, Colchester, Essex, United Kingdom; 4 Division of Management, Marketing and People, Business School, London South Bank University, London, United Kingdom; Columbia University, UNITED STATES

## Abstract

During the Covid-19 pandemic, universities in the UK used social media to raise awareness and provide guidance and advice about the disease to students and staff. We explain why some universities used social media to communicate with stakeholders sooner than others. To do so, we identified the date of the first Covid-19 related tweet posted by each university in the country and used survival models to estimate the effect of university-specific characteristics on the timing of these messages. In order to confirm our results, we supplemented our analysis with a study of the introduction of coronavirus-related university webpages. We find that universities with large numbers of students are more likely to use social media and the web to speak about the pandemic sooner than institutions with fewer students. Universities with large financial resources are also more likely to tweet sooner, but they do not introduce Covid-19 webpages faster than other universities. We also find evidence of a strong process of emulation, whereby universities are more likely to post a coronavirus-related tweet or webpage if other universities have already done so.

## Introduction

### University responses to the spread of respiratory illnesses

Pandemic outbreaks of respiratory illnesses have struck universities for hundreds of years. European universities have documented the effect of pandemics since at least the fourteenth century [1, 2]. Many American universities closed their campuses during the influenza pandemics of the twentieth century [3–7]. More recently, universities in Asia were severely affected by the 2009 H1N1 pandemic and the 2002–04 SARS outbreak.

Universities have incentives to prepare and respond to outbreaks of respiratory illnesses because they affect student health, reduce academic performance, and lead to increased use of health care [4, 6, 8–13].

In this context, universities’ first line of defence is influenza vaccination. While seasonal vaccination does not protect against the uncommon viruses at the heart of pandemics, they provide a basic level of protection [10] and reduce visits to doctors and health centres, as well as reduce hospitalisation. As a second line of defence against outbreaks of respiratory illnesses, universities implement non-pharmaceutical interventions, including isolation, social distancing, smothering of coughs and sneezes, washing hands, and cleaning touched objects and surfaces, among others [14–18].

Regardless of the specific interventions implemented to mitigate outbreaks of respiratory illnesses, universities must rely on timely and effective communication campaigns. In this light, we present a study of the timing of university communication during the height of the Covid-19 pandemic in the UK.

### University communication and social media during the Covid-19 pandemic: A crisis informatics approach to studying the impact of the pandemic on higher education

In early March 2020, Covid-19 had spread across the UK. At that time, the central government had not issued university-specific advice. Therefore, universities activated their response systems and implemented their own measures to control the disease on their campuses. In the first stage, universities raised awareness, reinforced public health advice, and provided guidance to students and staff [19]. Later on, they implemented more stringent measures, including social distancing and remote working for staff, particularly in mid-March 2020 when preparations for a national lockdown were in progress. In spite of some initial hesitation, universities closed their campuses to non-essential services by 23^rd^ March 2020. This variation in university responses to Covid-19 motivated us to look more closely at how universities reacted to the pandemic.

The initial information campaign on university campuses and the subsequent implementation of interventions were announced to students and staff through email and internal newsletters. These emails and newsletters are private tools of internal crisis communication and the research team did not have systematic access to them. Yet, part of this engagement was observable in universities’ social media channels, as universities are aware that students may prefer social media posts rather than emails [20], and this provided us with a unique opportunity to study how universities responded to the pandemic. Our investigation indicates that UK universities were making references to Covid-19 in social media since late January 2020. These social media posts generally raised awareness, reinforced public health advice, and provided guidance.

The public has been using social media and other forms of communication during crises to learn and inform themselves [21, 22]. Organisations have embraced social media to enable rapid interaction with stakeholders [23–26]. Universities also use social media to communicate with students and staff in a frequent, timely, open, and targeted manner [27–32].

The use of social media as a two-way communication channel between universities and students and staff during the Covid-19 pandemic places our research in the area of *crisis informatics* [33–39]. Crisis informatics is a relatively new field that explores the role of information and communication technology (ICT) in crises. Specifically, it focuses on how networked ICT facilitates the public’s response to a crisis. The field covers different types of crises, although it is particularly useful for the study of exogenous events such as natural hazards [37].

As the role of social media has become more important during crises, crisis informatics has made significant advances in several subjects, including the role of networked ICT on socio-behavioural factors during emergencies and the use of digital communication as a data source [37, 39, 40]. At the same time, there are challenges emerging from very large quantities of unstructured, noisy information. However, if the appropriate methods are applied to the collection, pre-processing, and analysis of data, social media can provide useful information for empirical analysis [37–39, 41, 42].

We rely on crisis informatics to contribute to the emerging research agenda on the impact of Covid-19 on higher education [43]. This research agenda, while fragmented and microscopic [44, 45], is making important contributions to our understanding of the effects of the SARS-CoV-2 virus and the pandemic on higher education. Currently, the emphasis has been on the disruption to traditional learning and the transition to online learning [46–50], as well as on the challenges in this transition, particularly for universities in developing countries [43, 51, 52].

Research has also been devoted to the timing and heterogeneity of non-pharmaceutical interventions during the height of the pandemic [53, 54]. Closely linked to this strand of work are epidemiological simulations for university campuses that inform university interventions, including contact tracing and quarantining [55, 56]. Interventions are supported by communication efforts and recent research has focused on communication strategies [20, 45, 51, 57–60], particularly on the use of social media and its positive effects on student satisfaction with university responses to the crisis [20, 45].

The pandemic not only affected students but also university staff, both physically and in terms of additional work pressure and general uncertainty. Thus, recent research has focused on the mental and physical health of staff [61, 62], and the key role of social support [61]. Recent work is also addressing the role of university leadership in managing the effects of the pandemic on campuses around the world and new studies are confirming the positive effect of women in managing the crisis [20, 63].

In summation, this paper explores the timing of coronavirus-related messages posted by universities in social media. Research shows that the timing of interventions can reduce the negative effects of pandemic outbreaks [64]. This is particularly pertinent to risk communication and therefore *our aim is to explain why some universities posted social media messages sooner than others*. In order to confirm our results, we supplemented our analysis of social media with a study of the introduction of coronavirus-related university webpages, which were also widely used by universities to communicate Covid-19 information to stakeholders [53].

### Theoretical framework

In order to explain variation in the timing of communication, we rely on Situational Crisis Communication Theory (SCCT) and theories of policy emulation.

During crises, organisations engage in strategic communication. According to Situational Crisis Communication Theory (65–66), institutions have strong incentives to communicate early with stakeholders when they are also victims of a crisis. This is often the case when natural disasters, including pandemics, take place–stakeholders do not attribute the crisis to the organisation, which in turn can benefit from providing information about the emergency. In fact, research evidence suggests that early communication by an organisation when a crisis is attributed to external factors contributes to the perceived credibility of the organisation [24, 65–68].

This logic is particularly important for UK universities in the context of the pandemic. According to SCCT, UK higher education institutions are victims of the pandemic and this gives them incentives to provide early information to their stakeholders in order to gain credibility. Institutional credibility was crucial because UK universities had to compete for students in the highly uncertain admission cycle of 2020. In this context of urgency and competition, our empirical analysis focuses on the variables that best reflect universities’ organisational capacity and ability to communicate early with students and staff.

Theories of policy emulation also help us understand the variation in the timing of university communications. While there are nuances across theories of emulation, they generally focus on the opportunities for policy diffusion: “Policy diffusion is the process whereby a state is more likely to adopt a policy if other states have already adopted that policy” [69]. We follow this literature and focus on the role of geographic proximity as a source of diffusion, which is best exemplified by Tobler’s first ‘law’ of geography where “everything is related to everything else, but near things are more related than distant things” [70]. More recent research adds a second ‘law:’ “Everything resembles everything else, but closer things are more similar” [70]. In terms of crisis communication, we expect that universities are more likely to communicate early with their stakeholders if universities in their vicinity have already done so.

In sum, our study contributes to our understanding of risk communication in the higher education sector during the pandemic and to our knowledge of the implementation of non-pharmaceutical interventions across campuses in the UK. These interventions, and the communication efforts that support them, are important because they slow down the spread of infection on campuses, thus reducing the negative effects of the pandemic on student health, academic performance, and use of health care. Moreover, and in the context of the pandemic in the UK, universities filled a vacuum caused by the absence of central government advice to higher education institutions. In so doing, universities were confirming their key role as public sources of trust and potentially reducing the negative effects of a decline of the higher education sector in the UK economy. Universities, as victims of the crisis, quickly engaged their stakeholders and raised awareness, reinforced public health advice, and provided guidance through social media, in order to meet their duty of care and gain credibility in an uncertain admissions cycle.

## Material and methods

In order to explain why some universities posted Covid-19-related social media messages sooner than others, we followed a two-fold strategy. First, we collected posts and their metadata from universities’ official Twitter accounts to identify the date of their first Covid-19-related tweet during the height of the Covid-19 pandemic. Second, we used these dates to estimate Cox survival models of elapsed time and survival models of diffusion to explore the role of emulation. We used these two types of models to explore whether universities choose the timing of communication based only on their university-specific characteristics or whether they also considered actions taken by other institutions.

To test the validity of our findings from Twitter data, we applied the research design described above to the dates of universities’ first official Covid-19 webpages.

### Twitter data

The crisis informatics literature explores several peer-to-peer communication platforms [35]. A large proportion of the research focuses on social media, including “blogging and microblogging, social networking sites, social media sharing platforms, and wikis” [42]. Although universities use multiple social media platforms, we focus on Twitter because most UK universities have a Twitter account. In addition, Twitter’s emphasis on text, as well as the wide availability of computational methods to pre-process Twitter content and analyse text as data, make it a suitable source of information for the analysis of risk communication. In this sub-section we describe how we identified universities’ first tweet with Covid-19 content.

As a first step, we focused on the Twitter accounts of all officially recognised universities and colleges in the UK as higher learning institutions that can award degrees [71]. This list includes 170 universities, although our sample consists of 166 universities because some institutions do not have a Twitter account, while others have ceased operations or their business model is mainly online teaching, which was not as severely affected by the pandemic. We manually reviewed the Twitter accounts used in this paper to confirm their authenticity. In addition, we replicated our analyses of Table 1 using only accounts verified by Twitter; these results are presented in S1 Table. Twitter verifies accounts that are determined to be in the public interest; this assures the public that these Twitter profiles are authentic.

**Table 1:** Cox models of days to first Covid-19 tweet.

	Model 1	Model 2	Model 3
Ln(Total Enrolment)	1.387***	1.397**	1.486***
	(0.153)	(0.188)	(0.189)
Proportion Income Tuition	0.487	0.474	0.365**
	(0.256)	(0.273)	(0.179)
Ln(Total Reserves)	1.294**	1.302**	
	(0.162)	(0.174)	
Ln(Public Interaction)	0.883**	0.879**	0.897*
	(0.0505)	(0.0540)	(0.0520)
Russell Group	1.424	1.451	1.563
	(0.514)	(0.559)	(0.562)
Buildings per capita		0.000148	
		(0.00150)	
Ln(Unrestricted Reserves)			1.131
			(0.143)
Observations	141	135	139
Subjects	141	135	139
Failures	139	133	137
Clusters	88	87	87
Log L	-550.7	-520.9	-542.3

Dependent variable: Days to first Covid-19 tweet. Event of interest: First Covid-19 tweet. Results in hazard ratios. Standard errors in parentheses clustered on UTLA. Oxford, Cambridge, and universities with negative total and negative unrestricted reserves are excluded from the analyses.

* *p* < 0.1

** *p* < 0.05

*** *p* < 0.01.

As a second step, we collected tweets posted between 31^st^ December 2019 –when the WHO first identified a statement from Wuhan Municipal Health Commission related to a new ‘viral pneumonia’–and the end of our study on 6^th^ May 2020. We used Twitter’s public API to collect tweets that provided data encoded in JavaScript Object Notation (JSON). The extraction produced 57,340 tweets for the 166 universities in our sample within our period of interest.

We focus on two attributes of tweets: the text content and the timestamp. The content of a tweet may contain non-textual characters, including URLs, mentions, hashtags, emojis, or numbers. We used text pre-processing methodologies to improve the quality of the data, mitigate the creative use of spacing and punctuation, and remove non-textual content. These methodologies include separating hyperlinks from the adjacent text, normalising Twitter-specific tokens (e.g., hashtags and URLs), extracting text from in between symbols, replacing ampersands, lowercasing the text, normalising multiple occurrences of vowels and consonants, normalising emojis and numbers, splitting numbers and emojis when adjacent to text, and removing non-alphanumeric characters. In general, we used text normalisation to produce text concordant with standard natural language processing approaches applied to formal text.

Once we pre-processed all tweets, we applied tokenisation to obtain a bag of words from each tweet. We then applied pattern-matching rules to extract tweets that mention the pandemic. Specifically, we used four keywords: ‘coronavirus’, ‘covid’, ‘COVID-19’, and ‘face-to-face.’ Our initial search had a more extensive set of keywords for pattern matching, but it produced a large set of irrelevant tweets. After some manual exploration, we found that these four keywords captured the most relevant tweets for the study; they are also a better reflection of the strict measures that universities would eventually implement, including the end of face-to-face teaching.

These pre-processing and tokenisation methods reduced our original sample of 57,340 tweets to 7,015 relevant tweets. We then simply ranked them by timestamp to select the first tweet of each university. We manually cross-checked the first tweet for each university and removed any results that produced a tweet that was not relevant to our search. Thus, our final sample includes the date of the first Covid-19 related tweet for 158 universities.

#### University-specific characteristics

We use survival analysis–also known as hazard analysis or event history modelling–to analyse why some universities posted Covid-19 related tweets sooner than others. This method focuses on time to an event or a transition. In biostatistics, for example, the emphasis may be on a patient’s time to death or remission after a cancer diagnosis [72]. In this paper, our event of interest is the first Covid-19 related tweet posted by a university. Thus, the dependent variable (*Days to Tweet*) is the number of days from 31^st^ December 2019 to the date of a university’s first Covid-19 related tweet. In our sample of 158 universities, 153 posted a Covid-19 related tweet; the remaining five universities did not post a first tweet by the end of our study and therefore we coded them as right-censored. Our data indicates that the median time to posting the first tweet is 66 days with a 95 per cent confidence interval of 62 to 72 days.

**Fig 1:**
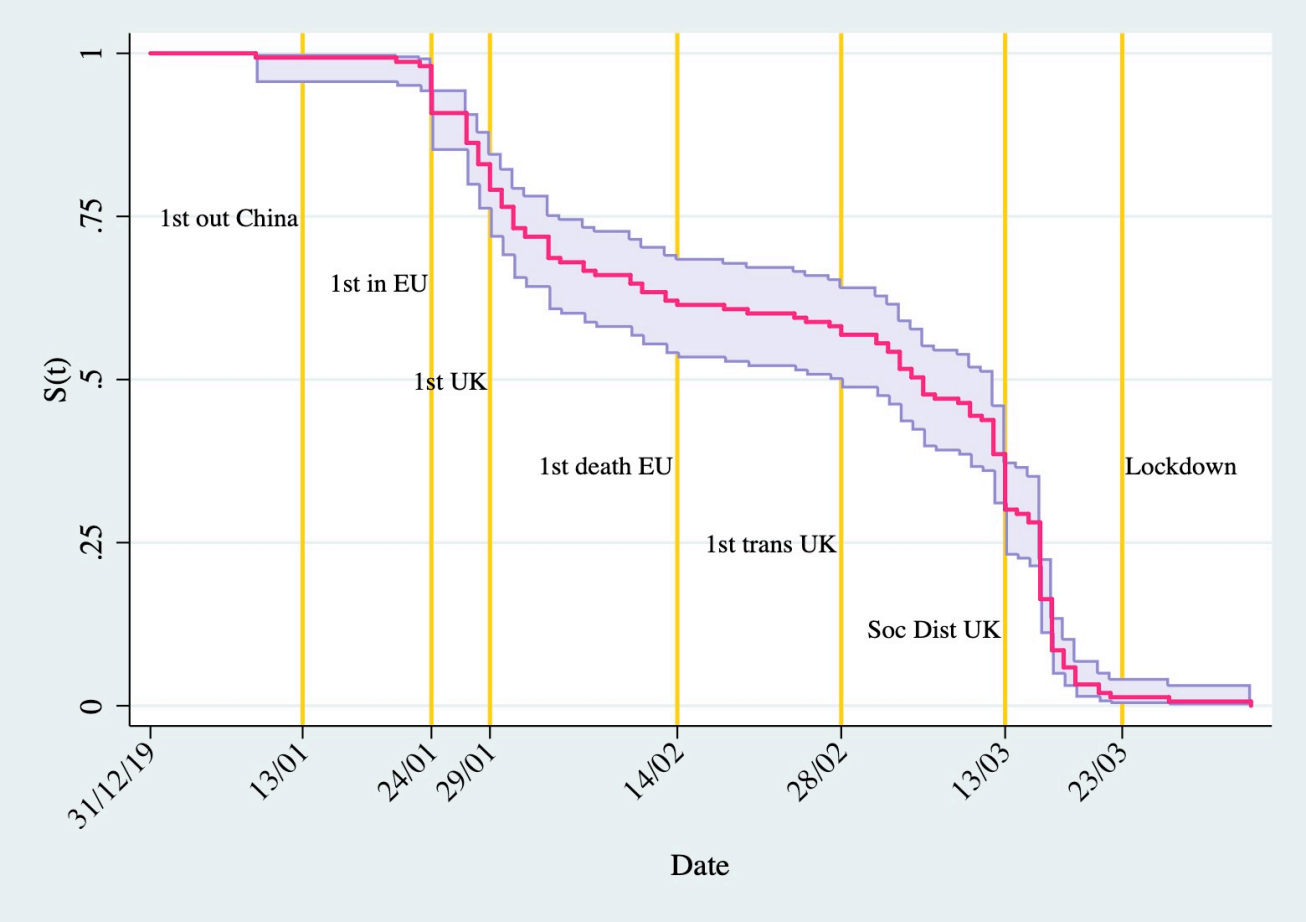
Survivor function of days to first Covid-19 tweet.

Fig 1 presents a more systematic analysis of the number of days to post the first tweet about Covid-19. The figure presents the Kaplan-Meier estimate of the survival function, which in this case can be interpreted as the proportion of universities that *have not posted* a Covid-19 tweet over time. On 31^st^ December 2019, not a single university had mentioned the novel coronavirus, but as time went by, more and more institutions posted a tweet about it. By 23^rd^ March, almost all universities in the UK had mentioned something about Covid-19 at least once.

There seem to be three periods in this graph. The first period is between 31^st^ December 2019 and 24^th^ January, when few universities posted their first tweet. In the second period, starting at the end of January, a larger number of universities posted their first Covid-19 related tweet, thus reducing the survival function drastically–by 28^th^ February 2020, when the first internal transmission was recorded in the UK, about 45 per cent of universities had already posted their first message. The third period starts in early March, when the preparations for strict non-pharmaceutical interventions were underway–by 13^th^ March, about 70 per cent of universities had posted their first tweet. By 23^rd^ March, almost all universities had posted at least one Covid-19 message on Twitter.

Why did some universities tweet sooner than others? In this section, we explore if universities choose the timing of their first tweet based *only* on their university-specific characteristics, such as the size of the student population or university financial resources. To the best of our knowledge, our paper presents the first analysis of the role of university-specific characteristics on the timing of communication during the Covid-19 pandemic.

First, we expect that universities with larger numbers of students will post a Covid-19 tweet sooner than universities with fewer students. We conjecture that most students and staff received official university messages about the pandemic over email or internal newsletters, but that there is a proportion of individuals who would not read those messages. For universities with a large number of students, that proportion could equate to thousands of individuals. In this case, posting messages and announcements in Twitter and other social media channels might be an effective way of reaching out to students and staff–messages are short and to the point, and can be re-posted by peers and colleagues, thus potentially reaching the students and staff who may not have read internal communications. In this case, posting a tweet sooner rather than later can be an effective way to raise awareness of the pandemic and provide guidance and advice to students and staff.

To measure the size of the student population in universities, we obtained the total number of student enrolments by higher education provider and applied a natural logarithm transformation to this number to produce the variable (*ln(Total Enrolment*)). This logarithmic transformation represents the orders of magnitude of student numbers and allows us to compare cases where some universities have more than 40,000 students and others have fewer than 500. We do not control for staff numbers because they are highly correlated with the size of the student population, thus creating a collinearity problem.

Our second set of expectations is related to resilience. The largest effect of the pandemic on UK universities will be caused by a decrease in student numbers [73]. In this light, our baseline model (Model 1) controls for additional university-specific factors that make universities more or less resilient to a negative shock to student numbers.

Our first control variable is the proportion of university income dependent on tuition fees. We expect that universities that rely heavily on tuition fees are more sensitive to a negative shock in student numbers than universities that are more research-oriented. The proportion of income dependent on tuition fees, which we label (*Proportion Income Tuition*), is simply the ratio of tuition fees to total income. Total income is composed of tuition fees, funding body grants, research grants, investment income, donations, and other income.

Our second control variable is university total reserves. Reserves are a measure of wealth and we expect that wealthy universities have the necessary resources to protect students and staff, and the capacity to endure a drastic reduction in student numbers. Total reserves are measured in millions of pounds sterling and include all types of university reserves, both restricted and unrestricted. As with the number of student enrolments, we applied a natural logarithm transformation to this variable to account for a large variation in the data; we labelled this variable (*ln(Total Reserves*)).

We excluded the University of Oxford and the University of Cambridge from all our analyses because they have financial resources that are incomparable to the resources of other universities, even when a logarithmic transformation is applied. Excluding Cambridge and Oxford, the mean total reserves for our sample of universities is £218 million. In contrast, Cambridge has £5.1 billion in total reserves while Oxford has £4.1 billion in reserves. We also removed from the analysis a very small number of universities that had negative total reserves.

Our third control variable is interaction with the public. This variable is measured as the number of attendants to free events, including lectures, performances, exhibitions, museums, and other events. As with the total number of student enrolments, we applied a natural logarithm transformation to account for a large variation in the data; we labelled this variable (*ln(Public Interaction)*). Interaction with the public is a double-edged sword, as it may increase the risk of infection through exposure but also strengthen resilience in terms of links to the community.

Our fourth control variable indicates whether a university is a member of the Russell Group of universities: (*Russell Group*). This variable is equal to one if a university is one of the 24 universities in the Russell Group and equal to zero otherwise. We expect that universities in this group will be more resilient because they are older–which provides experience in dealing with crises–but also because they have large financial resources and are research-intensive, which allows them to endure negative shocks to student numbers. We obtained the list of Russell Group universities from the group’s official website.

S2 Table presents additional analyses that control for the gender of university vice-chancellors and for the proportion of positions in university leadership teams occupied by women. As mentioned in the introduction, the characteristics of the leadership of an organisation play an important role on crisis response [20, 74, 75], and recent work on Covid-19 indicates that women are more effective in reducing Covid-19 deaths [63]. Results from S2 Table indicate that the gender of university vice-chancellors and the proportion of positions in university leadership teams occupied by women do not have a statistically significant effect on the timing of communication. The names and gender of university vice-chancellors were obtained from Universities UK [76] and from official university websites. The proportion of positions in university leadership teams occupied by women were obtained from official university websites.

To summarise, our baseline Model 1 of university-specific characteristics includes the *log(Total Enrolment*), *Proportion Income Tuition*, *Total Reserves*, *ln(Public Engagement)*, and *Russell Group* membership. In addition, we estimated two alternative models. Model 2 includes a measure of campus size as given by the number of university buildings per number of students and staff (*Buildings per capita*). Model 3 replaces *ln(Total Reserves*) with *ln(Unrestricted Reserves*). Unrestricted reserves, measured in millions of pounds sterling, are a component of total reserves but do not include sensitive sources of funds, such as a university’s endowment. We note again that we eliminated Oxford and Cambridge from all our analyses due to their enormous financial resources–the mean unrestricted reserves for our sample is £157 million. In contrast, Cambridge has over £3 billion in unrestricted reserves while Oxford has £2.8 billion. We also eliminated a handful of universities with negative unrestricted reserves.

The variables ln(Total Enrolment), Proportion Income Tuition, ln(Total Reserves), ln(Public Engagement), Buildings per capita, and ln(Unrestricted Reserves), were obtained from the Higher Education Statistics Authority (HESA) [77]. These variables correspond to the academic year 2018–19, with the exception of the number of buildings, which corresponds to the academic year 2017–18. These were the most recent statistics available from HESA when we completed our study and we believe that they have not changed drastically for the academic year 2019–20. Thus, they continue to provide an adequate reflection of university-specific characteristics during the height of the pandemic. Summary statistics for all variables for the estimation sample of our baseline Model 1 in Table 1 are presented in S3 Table. The specific tables from HESA used to support the findings of our study are presented in S1 Appendix.

We now turn to our estimation procedure. Table 1 presents three Cox-semiparametric models of our dependent variable *Days to Tweet*, which is the number of days from 31^st^ December 2019 to the date of a university’s first Covid-19 related tweet. All models in this paper were estimated in Stata version 15. We use Cox models because we do not have a strong theory about the shape of the hazard rate and therefore we prefer to leave it unparametrized. As long as the proportionality assumption is met by the models, this choice does not affect the substantive effects of our variables of interest.

We applied four different specifications of proportional hazards tests available in Stata 15 to all Cox models in this paper, including analysis time, the log of analysis time, one minus the Kaplan-Meier product-limit estimate, and the rank of analysis time [78]. All models passed either all four tests or at least two of them; we are confident that they meet the proportionality assumption. The tests are available in our replication files. If a model passed only two tests out of four, we decided not to adjust the non-proportional covariate because all variables in our Cox models are time-invariant and the proper solution to the problem is unlikely to bring large benefits while causing drastic changes to the research design [79].

The estimation results in Table 1 consist of hazard ratios–that is, exponentiated coefficients–and their standard errors clustered for the upper-tier local authority (UTLA) to address a potential lack of independence for universities within the same authority. An UTLA is a geographic unit in the UK often identical to a county, unitary authority, or London borough. For ease of interpretation of Table 1, a hazard ratio above one indicates an increase in the hazard rate–this is the rate at which universities post their first tweet over time since 31^st^ December 2019. In contrast, a hazard ratio below one indicates a decrease in the hazard rate. As an illustration, a hazard ratio of 1.3 indicates that a change in a covariate increases the hazard rate in 30 per cent, while a hazard ratio of 0.8 indicates a decrease of 20 per cent.

#### Emulation

In this section we investigate if universities consider the actions of other institutions in their decision to post a first Covid-19 related tweet. To do so, we estimate survival models of emulation used in the literature on public policy diffusion [80–87]. As mentioned in the introduction, “Policy diffusion is the process whereby a state is more likely to adopt a policy if other states have already adopted that policy” [69].

We do not aim to understand the causes of emulation–which may be connected to competition, for instance–but to look for evidence of a diffusion process across UK universities. To the best of our knowledge, this is the first study of diffusion in university communication during the Covid-19 pandemic.

Recent models of diffusion rely on dyadic data whereby pairs of states or countries are the unit of statistical analysis [82, 83, 88]. We follow this literature and use dyads of UK universities as units of analysis. For example, we create the dyad Essex-Bristol, Essex-Kent, Essex-Roehampton, and so on. For 170 universities, there are 170^2^ = 28,900 university dyads. Each of these dyads is followed daily from 31^st^ December 2019 to 6^th^ May 2020, which gives us a potential sample of 3,670,300 observations. Our sample is smaller because many universities posted their first tweet before the 6^th^ of May.

This daily dyadic setup for our data is useful because we can record the date when a university tweets for the first time and track if other universities have tweeted before in order to explore the likelihood of emulation. It is precisely for this reason that the dyad Essex-Kent is not the same as the dyad Kent-Essex: Kent may emulate Essex if Essex tweeted first, but Essex cannot emulate Kent.

In the daily dyad University A-University B, our dependent variable (*Emulation*) is equal to one on the day when University A posts its first tweet if University B has previously posted a tweet, and zero otherwise. The literature on diffusion prescribes that once *Emulation* takes on a value of one on a particular date, it should then be coded as missing; this is simply because we focus on time to emulation and because once two universities have taken the same course of action, that is, posting a tweet, emulation is no longer a possibility. For the estimation sample of Model 2 in Table 2, there are 5,831 cases where *Emulation* is equal to one and 853,141 cases where it is equal to zero.

**Table 2:** Models of first Covid-19 tweet and emulation of first Covid-19 tweet.

	Model 1:	Model 2:	Model 3:
	First Covid-19 tweet	Emulation of first Covid-19 tweet	Emulation of first Covid-19 tweet
	(monadic)	(dyadic unconditional)	(dyadic conditional)
Ln(Total Enrolment)_A_	1.440***	1.536***	1.514***
	(0.188)	(0.212)	(0.211)
Proportion Income Tuition _A_	0.750	0.211	0.235
	(0.649)	(0.204)	(0.229)
Ln(Total Reserves) _A_	1.328*	1.065	1.093
	(0.194)	(0.145)	(0.154)
Ln(Public Interaction) _A_	0.796**	0.919	0.915
	(0.0707)	(0.0682)	(0.0708)
Russell Group _A_	1.921	1.172	1.168
	(0.763)	(0.564)	(0.569)
Ln(Covid-19 Daily Cases) _A_	2.341***	1.505***	1.375**
	(0.390)	(0.239)	(0.215)
Days _A_	1.137***	0.828***	0.562***
	(0.0512)	(0.0363)	(0.0373)
Days^2^_A_	0.998*	1.005***	1.011***
	(0.000966)	(0.00109)	(0.00146)
Days^3^_A_	1.000**	1.000***	1.000***
	(0.00000575)	(0.00000717)	(0.00000915)
Ln(Total Enrolment)_B_		0.955***	0.980
		(0.0109)	(0.0131)
Proportion Income Tuition _B_		1.226**	2.243***
		(0.114)	(0.274)
Ln(Total Reserves) _B_		1.018	0.963*
		(0.0175)	(0.0190)
Ln(Public Interaction) _B_		1.009	1.024***
		(0.00670)	(0.00718)
Russell Group _B_		1.014	1.228***
		(0.0303)	(0.0489)
Ln(Covid-19 Daily Cases) _B_		1.413***	1.362***
		(0.0757)	(0.0653)
B Tweeted _A(t-2)_		27.91***	
		(7.157)	
(Neighbour)(B Tweeted _A(t-2)_)		0.646***	
		(0.0545)	
Neighbour			0.682***
			(0.0537)
Constant	0.000102***	0.000132***	3.957
	(0.000109)	(0.000126)	(4.227)
Observations	6930	858972	163670
Clusters	131	141	140
Pseudo-R2	0.167	0.352	0.175
Log L	-480.9	-22637.1	-20753.8

Dependent variable (Model 1): First Covid-19 tweet. Dependent variable (Models 2–3):

Emulation of first Covid-19 tweet. All models are discrete survival models with logit link and cubic polynomial for number of days to event. Results in odds ratios. Standard errors in parentheses clustered by university A. Oxford, Cambridge, and universities with negative total reserves are excluded from the analyses.

* *p* < 0.1

** *p* < 0.05

*** *p* < 0.01.

In a dyad, University A cannot emulate University B if the latter has not posted a tweet in the first place. Thus, our key determinant of emulation is a variable labelled (*B Tweeted*) that is equal to one if University B has tweeted and equal to zero otherwise. For example, in the dyad University A-University B, the former might tweet on 10^th^ March while the latter tweeted on 5^th^ March. For the estimation sample of Model 2 in Table 2, there are 171,382 cases where *B Tweeted* is equal to one and 687,590 cases where it is equal to zero. As prescribed in the literature on diffusion, the variable *B Tweeted* would then be equal to zero from 31^st^ December 2019 to 4^th^ of March and equal to one from 5^th^ March onwards. We do not expect that a tweet will have an immediate effect and therefore we use a two-day lag of this event. Our results are robust to the use of three and four-day lags for *B Tweeted*; S4 Table presents these additional estimation results.

Our dyadic data has all 28,900 dyads and a university may emulate any other university. Nevertheless, we expect that universities may be more responsive to the actions of their geographical neighbours because they share similar infection risks. Thus, we created a variable (*Neighbour*) that indicates whether two universities in a dyad are geographical neighbours. The variable *Neighbour* is equal to one if two universities are separated by a distance of 50 kilometres of less, and equal to zero otherwise. For the estimation sample of Model 2 in Table 2, approximately 12 per cent of universities are neighbours according to this definition.

In S5 Table, we present estimates from the dyadic models of Table 2 using two alternative definitions of geographic proximity. In the first alternative, two universities are neighbours if they are separated by a distance of 100 kilometres of less. In the second alternative, two universities are neighbours if they are separated by a distance of 25 kilometres of less. Our results are robust to these alternative definitions of a neighbourhood.

To calculate distances between universities, we used the Google Maps API to request the full address of each university, including its longitude and latitude. We then used these coordinates and the package ‘geodist’ [89] in *R* version 3.5.0 to create a matrix of distances for each dyad. We follow the literature on diffusion described above and interact the variable *Neighbour* with the variable *B Tweeted*. This interaction of variables allows us to investigate whether the likelihood of emulation depends on geographic proximity.

In addition to testing for the presence of diffusion, we use our research design to analyse the effect of the daily number of coronavirus infections in a university’s upper tier local authority. We focused on infection cases rather than deaths because the recording of Covid-19 related deaths in England is still a matter of debate. The number of Covid-19 cases was obtained from Public Health England as reported in Coronavirus (COVID-19) in the UK [90]. For the estimation sample of Model 2 in Table 2, the mean daily number of Covid-19 cases is 0.3 with a variance of 3.32; the minimum number is zero and the maximum is 33. We also applied a natural logarithm transformation to the number of Covid-19 cases and created the variable (*ln(Covid-19 Daily Cases)*). We collected this data on 30th April 2020 and therefore estimation is restricted to days between 31^st^ December 2019 and 30^th^ April 2020. This does not affect our analyses, as most universities had posted their first tweet by the end of March 2020.

The cases of Covid-19 are reported at the upper-tier local authority (UTLA) level in England. Unfortunately, these figures are not reported for Wales, Scotland, and Northern Ireland. However, we were able to include observations from universities in Wales, Scotland, and Northern Ireland until 30 January 2020, when there were no reported cases of infections in the UK. There are efforts to collect and organise coronavirus cases for Scotland and Wales using medical wards [91], but these are not comparable to the UTLAs in England.

We matched universities to UTLAs using their coordinates as explained above and assigning them to the polygons of UTLAs. These polygons were obtained from the Office of National Statistics file on Counties and Unitary Authorities (December 2017) Full Clipped Boundaries in UK [92]. We used the package ‘sp’ [93] in R version 3.5.0 to assign university coordinates to UTLA polygons.

Our models of diffusion also control for all the university-specific variables used in the previous section. Although these variables do not change between 31^st^ December 2019 and 6^th^ May 2020, they are useful indicators of university-specific characteristics. Our controls include university total reserves, and therefore we exclude Oxford, Cambridge, and universities with negative total reserves from our analyses of emulation.

In dyadic models, it is also recommended that specifications include control variables for both University A and University B [83]. This is simply because the probability of emulation depends on the actions of the two universities: the leader and the follower. Thus, all specifications include controls for both universities in a dyad, which we separate with subscripts. For instance, Model 2 in Table 2 controls for the natural logarithm of total student enrolments in University A, denoted, Ln(Total Enrolment)_A_, and for the natural logarithm of total student enrolments in University B, denoted, Ln(Total Enrolment)_B_.

We note that the literature on diffusion finds that traditional dyadic models create a bias in favour of an emulation effect. The intuition behind the bias is as follows: “Simply put, state *i* appears to emulate state *j* not because it looks to state *j* as a policy leader, but because both are independently headed in the same direction and state *j* may just happened to get there first.” [83] In other words, the traditional dyadic model cannot distinguish if variables increase the likelihood that University B will implement a policy (and therefore that there is an opportunity for emulation) or if they increase the probability that University A will emulate University B. The solution to this bias is quite simple; rather than estimating the original, unconditional dyadic model, one needs to estimate a model that conditions on a university’s opportunity to emulate. In this light, the purpose of the conditional model is not to find evidence of emulation but to distinguish if specific variables have an effect on emulation or on coincidental convergence.

In practical terms, in the conditional dyadic setup, the dependent variable is also *Emulation*, but the estimation sample is restricted to those days when there is an opportunity for emulation, that is, those days after University B has posted its first tweet. Thus, we condition on the variable (*Opportunity*), which is equal to one if University B has tweeted and equal to zero otherwise. For the estimation sample of Model 2 in Table 2, there are 163,670 cases where *Opportunity* is equal to one and 695,302 cases where it is equal to zero. In the dyadic conditional model where estimation is restricted to the 163,670 cases where *Opportunity* is equal to one, there are 5,831 cases where *Emulation* is equal to one and 157,839 cases where it is equal to zero. We note that the variable Opportunity is not identical to the variable B Tweeted because the opportunity to emulate starts the day after University B has tweeted.

Table 2 presents three models: a monadic survival model of universities’ first tweet, a dyadic unconditional model of emulation, and a conditional model of emulation. The goal of the first model is to explore the effect of Covid-19 cases on the hazard rate of posting a first Covid-19 related tweet. The previous section did not explore the effect of infections simply because it uses a cross-section of universities, while the data for infections is measured daily. Thus, it was more appropriate to present this test here because it uses the same daily data organisation than the dyadic models. Having said this, the purpose of Model 2 in Table 2 is to look for evidence of emulation. Model 3 is the conditional model of emulation and its goal is to differentiate the effect of variables in the likelihood of emulation or coincidental convergence.

Lastly, we note that all models in Table 2 are discrete survival models [94, 95]. Discrete survival models are implemented as models for binary choice–in our case, a logit model–that controls for duration dependence by adding a cubic polynomial of days between 31^st^ December 2019 and the event of interest [95]. In our case, the event of interest in Model 1 is a university’s first tweet, while in Models 2 and 3 the event of interest is emulation. Results for all models are presented in odds ratios. Standard errors clustered at University A in the dyad University A-University B are presented in parentheses in order to account for a potential lack of independence among observations. As in the previous section, we excluded the University of Oxford and the University of Cambridge, as well as any universities with negative total or unrestricted reserves.

### Webpages data

In this section we supplement our analysis of Twitter data with information from university webpages. Universities also used Covid-19 dedicated webpages to raise awareness of the pandemic and provide guidance and advice to students and staff [53]. We acknowledge that the content of Covid-19 specific webpages is different than Twitter posts–webpages require more careful planning and implementation than tweets, as well as constant updating and maintenance. It is precisely for this reason that an analysis of webpages is important, as any confirmation of substantive results will give more confidence to the analysis presented in the previous section.

Our research design is the same as in our analysis of Twitter data. We explored if universities introduce Covid-19 webpages based on their own factors *and* the actions taken by other universities. We also used the same estimation methods. First, we used Cox models for the analysis of the number of days to posting a first webpage, as well as the same university-specific control variables. Second, we used models of diffusion and controlled for the same time-varying variables as in the previous section, including the introduction of webpages by other universities and the number of Covid-19 cases in a university’s UTLA.

We began by identifying the date when universities first introduced a webpage with Covid-19 related information. We first mapped UK universities to their corresponding web domains, for instance essex.ac.uk. We then used the Google Search API to search every domain from 31^st^ December 2019 to 6^th^ May 2020 for the Covid-19 related keywords: ‘Covid-19’, ‘Corona,’ and ‘Coronavirus.’ The returned results for each matching page included a summary snippet, a title, and a Uniform Resource Locator (URL).

Unlike tweets, these webpages are as noisy as they are heterogenous in design, and a one-size-fits-all approach to noise reduction would not be useful to extract content. Therefore, our text extraction was limited to the page body, which allowed us to focus on the main text in a webpage while limiting noise in the navigation menus or announcements that contain Covid-19 related terms. This process produced 13,265 matching webpages for 128 universities.

**Fig 2:**
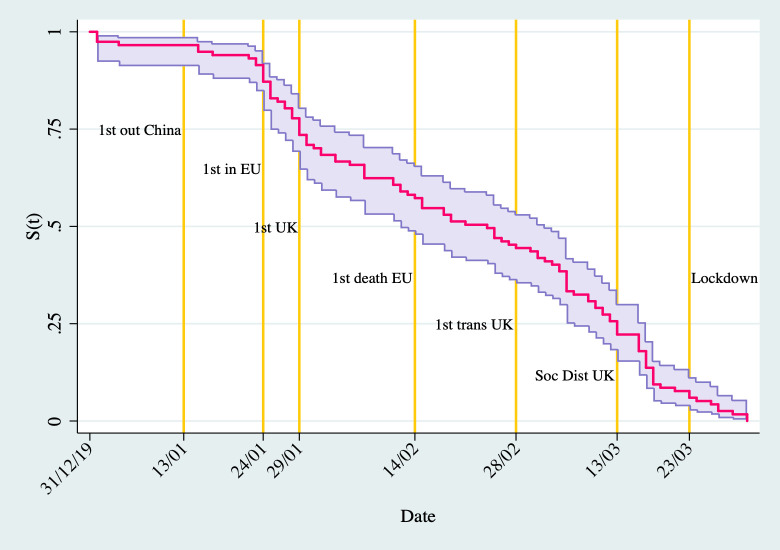
Survivor function of days to first Covid-19 webpage.

We sorted these matching webpages by date and manually inspected the top result for each university to minimise noise. We used the dates from these webpages to produce the dependent variable (*Days to Webpage*), which is the number of days from 31^st^ December 2019 to the date of a university’s first Covid-19 webpage as described above. We note that we do not have any right-censored cases because our data collection produced a sample of 128 universities with a webpage. Unfortunately, we cannot be sure that the remaining 42 universities in the UK did not introduce a Covid-19 webpage and therefore it would be incorrect to code them as right-censored. Having said this, our data indicates that the median time to posting the first webpage is 55 days with a 95 per cent confidence interval of 43 to 63 days. Fig 2 presents the Kaplan-Meier estimate of the survival function of the number of days to introduce a webpage about Covid-19.

We now turn to our estimation strategy. For the Cox models, our dependent variable is the number of days from 31^st^ December 2019 to the date when a university first introduced a Covid-19 webpage.

Table 3 presents three Cox-semiparametric models of our dependent variable (*Days to Webpage*). As in the previous section, we use Cox models because we do not have a strong theory about the shape of the hazard rate and therefore we prefer to leave it unparametrized. Likewise, the estimation results in Table 3 consist of hazard ratios with their standard errors clustered for the upper-tier local authority (UTLA) presented in parentheses.

**Table 3:** Cox models of days to first Covid-19 webpage.

	Model 1	Model 2	Model 3
Ln(Total Enrolment)	1.340***	1.482***	1.361***
	(0.150)	(0.220)	(0.153)
Proportion Income Tuition	0.188***	0.254***	0.175***
	(0.0999)	(0.128)	(0.0923)
Ln(Total Reserves)	1.058	1.079	
	(0.139)	(0.154)	
Ln(Public Interaction)	0.982	1.004	0.987
	(0.0525)	(0.0506)	(0.0526)
Russell Group	1.134	1.027	1.286
	(0.429)	(0.395)	(0.474)
Buildings per capita		271972.2	
		(2475205.5)	
Ln(Unrestricted Reserves)			0.980
			(0.108)
Observations	111	106	109
Subjects	111	106	109
Failures	111	106	109
Clusters	77	76	76
Log L	-409.0	-384.7	-400.0

Dependent variable: Days to first Covid-19 webpage. Results in hazard ratios. Standard errors in parentheses clustered on UTLA. Oxford, Cambridge, and universities with negative total and unrestricted reserves are excluded from the analyses.

* *p* < 0.1

** *p* < 0.05

*** *p* < 0.01.

We now turn to our analysis of emulation, which used the same specifications as the emulation models of Twitter data, although the key determinant of emulation in this section is a variable labelled (*B Webpage*) that is equal to one if University B has introduced a Covid-19 webpage and equal to zero otherwise. For the estimation sample of Model 2 in Table 4, there are 99,214 cases where *B Webpage* is equal to one and 307,954 cases where it is equal to zero.

**Table 4:** Models of first Covid-19 webpage and emulation of first Covid-19 webpage.

	Model 1:	Model 2:	Model 3:
	First Covid-19 webpage	Emulation of first Covid-19 webpage	Emulation of first Covid-19 webpage
	(monadic)	(dyadic unconditional)	(dyadic conditional)
Ln(Total Enrolment)_A_	0.952	1.277	1.276
	(0.145)	(0.218)	(0.217)
Proportion Income Tuition _A_	0.203*	0.677	0.678
	(0.185)	(0.774)	(0.774)
Ln(Total Reserves) _A_	1.338*	1.207	1.211
	(0.208)	(0.231)	(0.232)
Ln(Public Interaction) _A_	1.015	1.004	1.004
	(0.0565)	(0.0526)	(0.0524)
Russell Group _A_	0.862	0.724	0.721
	(0.537)	(0.347)	(0.349)
Ln(Covid-19 Daily Cases) _A_	1.921**	1.616*	1.600*
	(0.612)	(0.402)	(0.396)
Days _A_	1.203**	1.102	1.034
	(0.101)	(0.0694)	(0.0670)
Days^2^_A_	0.997*	0.998	0.999
	(0.00185)	(0.00136)	(0.00138)
Days^3^_A_	1.000*	1.000	1.000
	(0.0000123)	(0.00000850)	(0.00000856)
Ln(Total Enrolment)_B_		0.996	1.004
		(0.0115)	(0.0109)
Proportion Income Tuition _B_		0.882***	0.931*
		(0.0428)	(0.0373)
Ln(Total Reserves) _B_		1.023**	1.006
		(0.00932)	(0.00961)
Ln(Public Interaction) _B_		0.989**	0.989**
		(0.00410)	(0.00424)
Russell Group _B_		1.009	0.984
		(0.0134)	(0.00968)
Ln(Covid-19 Daily Cases) _B_		1.076	1.070
		(0.0617)	(0.0605)
B Webpage _A(t-2)_		30.79***	
		(4.353)	
(Neighbour)(B Webpage _A(t-2)_)		0.837**	
		(0.0748)	
Neighbour			0.849*
			(0.0725)
Constant	0.000691***	0.00000388***	0.000426***
	(0.00111)	(0.00000558)	(0.000625)
Observations	4061	407168	95459
Clusters	81	109	109
Pseudo-R2	0.136	0.327	0.127
Log L	-312.4	-13252.3	-12821.1

Dependent variable (Model 1): First Covid-19 webpage. Dependent variable (Models 2–3):

Emulation of first Covid-19 webpage. All models are discrete survival models with logit link and cubic polynomial for number of days to event. Results in odds ratios. Standard errors in parentheses clustered by university A. Oxford, Cambridge, and universities with negative total reserves are excluded from the analyses.

* *p* < 0.1

** *p* < 0.05

*** *p* < 0.01.

We estimated three models: one monadic model of universities’ first Covid-19 related webpage, and two dyadic models of emulation, one unconditional and one conditional. For the estimation sample of Model 2 in Table 4, there are 3,405 cases where *Emulation* is equal to one and 403,763 cases where it is equal to zero. In the same sample, there are 95,459 cases where *Opportunity* is equal to one and 311,709 cases where it is equal to zero. Conditioning the analysis to the 95,459 cases where *Opportunity* is equal to one, there are 3,405 cases where *Emulation* is equal to one and 92,054 cases where it is equal to zero.

Table 4 presents results in odds ratios, which reflect changes in the odds of posting a first Covid-19 related webpage in Model 1 and the odds of emulation in Models 2–3. Standard errors clustered at University A in dyad University A-University B are presented in parentheses in order to account for a potential lack of independence among observations. As in the previous section, we excluded the University of Oxford and the University of Cambridge, as well as any universities with negative total or unrestricted reserves.

Our results are robust to the use of three and four-day lags for *B Webpage* for Model 2 in Table 4 (results presented in S6 Table), and to alternative definitions of a neighbourhood in the dyadic models of Table 4 (results presented in S7 Table).

## Results and discussion

We organise our discussion around our two sets of results. First, we discuss the effects of the size of the student community, the role of universities’ financial resources, and the impact of Covid-19 infections on the hazard rate of posting a first Covid-19 related tweet. We then consider if evidence from our analysis of university webpages supports our conclusions. Second, we discuss the role of emulation and whether estimation results are consistent across our two sources of data.

In order to guide our discussion, we focus on the hazard ratios of independent variables, and particularly if they are above one (increase hazard rates) or below one (decrease hazard rates), at an alpha level of 0.05. For consistency, we apply the same terminology to Cox models and our discrete survival models with logits links.

### University size, financial wealth, and infections

Our first expectation is related to the size of the student community. We conjectured that not all university students and staff read university Covid-19 announcements communicated via email and internal newsletters. In fact, students may prefer social media posts rather than emails [20]. Therefore, universities have incentives to reinforce these announcements through social media; these incentives are stronger in large institutions simply because the number of individuals who may not have read private messages is larger. Thus, we expected that the number of student enrolments would increase the hazard rate of posting a first Covid-19 tweet. The hazard ratios for Ln(Total Enrolment) in the Cox models of Table 1 and the monadic model of Table 2 are well above one and statistically significant. This indicates that changes to the natural logarithm of total enrolment–which can also be interpreted as the elasticity of enrolment or per cent changes in total enrolment–increase the hazard rate of posting a tweet. *In other words*, *universities with larger numbers of students tweeted sooner than universities with fewer students*. This effect is also present in our analyses of universities with verified Twitter accounts, presented in S1 Table.

Our second set of expectations focuses on university-specific characteristics that determine resilience to a negative shock in student numbers. While there are multiple characteristics that deserve discussion, we highlight the role of financial resources because we expect that they will increase university resilience in the same way that countries’ wealth strengthens disaster preparedness and response [96–98].

The hazard ratios for Ln(Total Reserves) in the Cox models of Table 1 are well above one and statistically significant, which indicates that per cent changes in total reserves increase the hazard rate of posting a Covid-19 related tweet. While the monadic model of Table 2 indicates that the hazard ratio for Ln(Total Reserves) is significant only at an alpha level of 0.1, our analyses of universities with verified Twitter accounts in Table 2 confirm that wealth increases the hazard rate. *Altogether*, *we find that wealthier universities were more likely to tweet sooner than universities with more modest means*.

In the context of the current pandemic, we explored the effect of the number of Covid-19 cases on the hazard rate of posting a Covid-19 related tweet. To do so, we used the daily number of infections in universities’ upper-tier local authority as a control variable in our monadic model of universities’ first Covid-19 related tweet in Table 2. The hazard ratio for Ln(Covid-19 Daily Cases)_A_ in this model is well above two and statistically significant, which indicates that *per cent changes in Covid-19 cases greatly increase the hazard rate of posting a first Covid-19 tweet*.

We also note that the hazard ratio for Ln(Covid-19 Daily Cases)_A_ in the dyadic models of Table 2 are also above one and significant, which indicates that Covid-19 infections also increase the likelihood of emulation. The fact that the coefficients for Ln(Covid-19 Daily Cases)_A_ are quite similar across the unconditional and conditional dyadic models suggests that infections are driving emulation and not coincidental convergence.

We now consider if the effects of the size of the student community, the role of universities’ financial resources, and the impact of Covid-19 infections in our Twitter data are also present in our analyses of university webpages.

We acknowledged that data from Twitter can be quite noisy and therefore we supplemented our analyses with information from official university Covid-19 webpages. We identified the date when universities first introduced a Covid-19 webpage and then applied the same research design implemented for our Twitter data to estimate survival models and models of diffusion. To summarise these results, *the analyses from webpages provide moderate support for the effect of university size and indicate that university financial resources do not have a statistically significant effect on the hazard rate of introducing a webpage*. *Nonetheless*, *these analyses confirm the effect of Covid-19 infections on the odds of introducing a first Covid-19 webpage*.

First, the hazard ratios for Ln(Total Enrolment) in the Cox models of Table 3 are well above one and statistically significant, which indicates that per cent changes in student enrolments increase the hazard rate of introducing a Covid-19 webpage. However, the monadic model of a first Covid-19 webpage in Table 4 indicates that student enrolments do not have a significant effect on the rate of introducing a webpage. We consider that this is only moderate support for the effect of the size of the student community on the hazard rate of introducing a webpage.

Moreover, the models do not find support for an effect of university financial resources. In fact, all Cox models in Table 3 find that Ln(Total Reserves) does not have a statistically significant effect, while the monadic model of a first Covid-19 webpage in Table 4 indicates that university resources would increase the hazard rate of introducing a webpage only at an alpha level of 0.1. This suggests that university reserves do not determine the likelihood of introducing a Covid-19 webpage.

Nevertheless, our analyses of webpage data confirm the effect of Covid-19 infections on the timing of risk communication. Indeed, the hazard ratio for Ln(Covid-19 Daily Cases)_A_ in the monadic model of universities’ first webpage in Table 4 is well above one and statistically significant, which indicates that per cent changes in Covid-19 cases increase the hazard rate of tweeting. We also observed this effect in our analysis of universities’ first Covid-19 tweet.

It is also important to note that the hazard ratio for Ln(Covid-19 Daily Cases)_A_ in the dyadic models of Table 4 is also above one and significant, which indicates that Covid-19 infections also increase the likelihood of emulation. As with Twitter data, the coefficients for Ln(Covid-19 Daily Cases)_A_ are very similar across the unconditional and conditional dyadic models, which suggests that infections are driving emulation and not coincidental convergence.

### Emulation

One of the central features of our research design is the estimation of models of diffusion. We estimated conditional and unconditional dyadic models of emulation to explore whether universities choose the timing of communication based only on their own university-specific characteristics or whether the actions of other universities also contributed to their response. As mentioned, we do not aim to understand the causes of emulation but to look for evidence of a diffusion process across UK universities.

Our unconditional dyadic model of emulation in Table 2 indicates that the hazard ratio for B Tweeted _A(t-2)_ is very well above one and statistically significant. This suggests that universities are much more likely to follow institutions that have previously posted a Covid-19 related tweet. This effect is also present when we use three and four-day lags for B Tweeted, as indicated in our supplementary analyses in S4 Table. Evidence of emulation is one of the strongest results in our analyses and it is also replicated in our study of university webpages.

Interestingly, while a follower’s likelihood of emulation is higher when other universities have posted a tweet, this likelihood is not as high if the leading university is a geographical neighbour, as demonstrated by the hazard ratio for (Neighbour)(B Tweeted _A(t-2)_) in Table 2, which is smaller than one and statistically significant. We confirmed this effect in our supplementary analyses in S5 Table, which use two alternative definitions of a neighbourhood.

Our analyses of university webpages strongly confirm that universities are more likely to emulate if other institutions have previously posted a Covid-19 related webpage. Results from Table 4 indicate that the hazard ratio for B Webpage _A(t-2)_ is also very well above one and statistically significant. The results are of the same magnitude, direction, and significance as in our analyses of Twitter data–this is a very strong indication of the effect of diffusion in university responses during the pandemic. Moreover, this effect is also present when we use three and four-day lags for B Webpage, as indicated in our supplementary analyses in S6 Table. They also confirm that while a follower’s likelihood of emulation is higher when other universities have posted a webpage, this likelihood is not as high if the leading institution is a geographical neighbour, even when different definitions for a neighbourhood are used for estimation, as demonstrated in S7 Table.

These results point to a form of inequality among universities in the UK. Our estimation results indicate that universities with large student communities are quicker to engage in risk communication as measured by the timing of their first Covid-19 tweet and their first Covid-19 webpage. While all universities have similar incentives to reach out sooner to larger numbers of students during crises, the ability to do so depends on wealth. It is therefore not a coincidence that our estimation results suggest that universities with large financial resources, as measured by total reserves, are also quicker to engage in risk communication over social media.

Universities with large student communities and vast financial resources have something else in common: age. In the UK, a university’s age is crucial because it brings wealth and experience with previous crises, and research shows that this has a positive effect in prevention [96, 99]. This simply means that older universities are wealthier, larger, and more experienced, and altogether more resilient to pandemics. These characteristics allow them to engage in risk communication at an earlier stage than other universities. Smaller, poorer, younger universities are not so resilient and this is reflected in the timing of their risk communication, which lags behind the efforts of more established universities. This coincides with the finding by the Institute of Fiscal Studies that universities with weak financial positions before the pandemic are at higher risk of insolvency as a result of the shock to student numbers [73].

On the more positive side, our analyses show that universities learn from each other. This means that there is a space for leadership and an opportunity for coordination during crises. While some coordination was organised by Universities UK, in terms of the negative consequences of the pandemic on universities’ financial positions, there is a need for better coordination in the delivery of risk communication and the sharing of best practice that can allow the system to learn more quickly and respond more effectively to crises.

Indeed, a more effective crisis response would reduce the negative effects of the pandemic on the education sector and its link to the national economy. The UK education sector produces close to six per cent of national output and in the second quarter of 2020 it was estimated that 90 per cent of this output would be lost due to the pandemic [100]. At that time, multiple studies predicted that UK universities would lose billions of pounds in the long run and that some institutions would not be financially viable without significant government assistance [73, 100]. Our study shows that UK universities engaged in swift crisis communication in the absence of central government guidelines, which probably reduced some of the negative consequences of the pandemic.

In this light, we draw important lessons for universities around the world and contribute to our general understanding of the effects of the pandemic on higher education. Although the empirical results are only valid for institutions in the UK, the paper provides a useful research design that can be replicated for data on university responses in other countries [53, 59]. In addition, our theoretical framework and selection of covariates, as well as the emphasis on survival analysis and models of policy diffusion, will serve as useful guidelines for further research.

## Supporting information

S1 Table Verified Twitter accounts. (DOCX)

S2 Table Additional controls for university leadership for Model 1 in Table 1 and Model 1 in Table 3. (DOCX)

S3 Table Summary statistics. (DOCX)

S4 Table Additional lags for Model 2 in Table 2 (dyadic unconditional). (DOCX)

S5 Table Alternative definitions of a neighbourhood for dyadic models of Table 2. (DOCX)

S6 Table Additional lags for Model 2 in Table 4 (dyadic unconditional). (DOCX)

S7 Table Alternative definitions of a neighbourhood for dyadic models of Table 4. (DOCX)

S1 Appendix Specific HESA tables. (DOCX)
