# Supplementary material for: Variation in the timing of Covid-19 communication across universities in the UK
Source: PLoS One. 2021 Feb 16;16(2):e0246391. doi: 10.1371/journal.pone.0246391 (PMC7886223; doi:10.1371/journal.pone.0246391)
Supplement: S1 Table — (DOCX) [file pone.0246391.s001.docx]

**S1 Table. Verified Twitter accounts**

|  | Model 1 | Model 2 | Model 3 |
| --- | --- | --- | --- |
| Ln(Total Enrolment) | 1.393*** | 1.407** | 1.569*** |
|  | (0.167) | (0.224) | (0.274) |
| Proportion Income Tuition | 0.653 | 0.662 | 0.467 |
|  | (0.424) | (0.443) | (0.278) |
| Ln(Total Reserves) | 1.427*** | 1.424** |  |
|  | (0.188) | (0.201) |  |
| Ln(Public Interaction) | 0.843** | 0.845** | 0.863** |
|  | (0.0618) | (0.0628) | (0.0599) |
| Russell Group | 1.377 | 1.348 | 1.509 |
|  | (0.548) | (0.533) | (0.601) |
| Buildings per capita |  | 3.109 |  |
|  |  | (28.94) |  |
| Ln(Unrestricted Reserves) |  |  | 1.187 |
|  |  |  | (0.165) |
| Observations | 109 | 107 | 107 |
| Subjects | 109 | 107 | 107 |
| Failures | 108 | 106 | 106 |
| Clusters | 76 | 75 | 75 |
| Log L | -397.9 | -389.5 | -390.4 |

Dependent variable: Days to first Covid-19 tweet. Event of interest: First Covid-19 tweet. Results in hazard ratios. Standard errors in parentheses clustered on UTLA. Oxford, Cambridge, and universities with negative total and negative unrestricted reserves are excluded from the analyses.

* *p* < 0.1, ** *p* < 0.05, *** *p* < 0.01
