# Supplementary material for: Variation in the timing of Covid-19 communication across universities in the UK
Source: PLoS One. 2021 Feb 16;16(2):e0246391. doi: 10.1371/journal.pone.0246391 (PMC7886223; doi:10.1371/journal.pone.0246391)
Supplement: S2 Table — (DOCX) [file pone.0246391.s002.docx]

**S2 Table. Additional controls for university leadership for Model 1 in Table 1 and Model 1 in Table 3**

|  | **First Covid-19 Tweet (Table 1)** | | **First Covid-19 Webpage (Table 3)** | |
| --- | --- | --- | --- | --- |
|  | Model 1 | Model 2 | Model 3 | Model 4 |
| Ln(Total Enrolment) | 1.390*** | 1.354*** | 1.343*** | 1.392*** |
|  | (0.153) | (0.140) | (0.153) | (0.166) |
| Proportion Income Tuition | 0.447 | 0.399 | 0.198*** | 0.191*** |
|  | (0.239) | (0.251) | (0.104) | (0.0982) |
| Ln(Total Reserves) | 1.295** | 1.333*** | 1.059 | 1.024 |
|  | (0.159) | (0.138) | (0.142) | (0.140) |
| Ln(Public Interaction) | 0.890** | 0.887** | 0.986 | 0.999 |
|  | (0.0500) | (0.0502) | (0.0478) | (0.0478) |
| Russell Group | 1.372 | 1.298 | 1.164 | 1.202 |
|  | (0.498) | (0.456) | (0.418) | (0.428) |
| VC Gender | 1.213 | 1.195 | 0.780 | 0.777 |
|  | (0.216) | (0.232) | (0.189) | (0.195) |
| Proportion women in exec team |  | 0.787 |  | 0.872 |
|  |  | (0.482) |  | (0.475) |
| Observations | 141 | 138 | 111 | 107 |
| Subjects | 141 | 138 | 111 | 107 |
| Failures | 139 | 136 | 111 | 107 |
| Clusters | 88 | 87 | 77 | 75 |
| Log L | -550.2 | -535.4 | -408.3 | -389.2 |

Dependent variable: Days to first Covid-19 tweet. Event of interest: First Covid-19 tweet. Results in hazard ratios. Standard errors in parentheses clustered on UTLA. Oxford, Cambridge, and universities with negative total and negative unrestricted reserves are excluded from the analyses. For the estimation sample of Model 1, the proportion of women VCs is 30.5% while the mean proportion of positions occupied by women in an executive team is 0.402. For the estimation sample of Model 3, the proportion of women VCs is 25.2% while the mean proportion of positions occupied by women in an executive team is 0.394.

* *p* < 0.1, ** *p* < 0.05, *** *p* < 0.01
