# Supplementary material for: Variation in the timing of Covid-19 communication across universities in the UK
Source: PLoS One. 2021 Feb 16;16(2):e0246391. doi: 10.1371/journal.pone.0246391 (PMC7886223; doi:10.1371/journal.pone.0246391)
Supplement: S3 Table — (DOCX) [file pone.0246391.s003.docx]

**S3 Table. Summary statistics**

|  | N | Mean | Variance | Min | Max |
| --- | --- | --- | --- | --- | --- |
| Ln(Total Enrolment) | 141 | 9.18 | 1.25 | 5.58 | 10.63 |
| Proportion Income Tuition | 141 | 0.59 | 0.04 | 0.02 | 0.88 |
| Ln(Total Reserves) | 141 | 4.76 | 1.46 | 0.77 | 7.63 |
| Ln(Public Interaction) | 141 | 10.81 | 4.38 | 6.52 | 19.25 |
| Russell Group | 141 | 0.14 | 0.12 | 0 | 1 |
| Buildings per capita | 135 | 0.01 | 0.00 | 0 | 0.08 |
| Ln(Unrestricted Reserves) | 139 | 4.36 | 1.66 | 0.77 | 7.20 |
