# Supplementary material for: Variation in the timing of Covid-19 communication across universities in the UK
Source: PLoS One. 2021 Feb 16;16(2):e0246391. doi: 10.1371/journal.pone.0246391 (PMC7886223; doi:10.1371/journal.pone.0246391)
Supplement: S5 Table — (DOCX) [file pone.0246391.s005.docx]

**S5 Table. Alternative definitions of a neighbourhood for dyadic models of Table 2**

|  | Emulation of first Covid-19 tweet  (dyadic unconditional)  Neighbor far | Emulation of first Covid-19 tweet  (dyadic unconditional)  Neighbor close | Emulation of first Covid-19 tweet  (dyadic conditional)  Neighbor far | Emulation of first Covid-19 tweet  (dyadic conditional)  Neighbor close |
| --- | --- | --- | --- | --- |
| Ln(Total Enrolment)_A_ | 1.557*** | 1.539*** | 1.534*** | 1.515*** |
|  | (0.215) | (0.214) | (0.214) | (0.212) |
| Proportion Income Tuition _A_ | 0.207 | 0.210 | 0.230 | 0.234 |
|  | (0.200) | (0.203) | (0.226) | (0.228) |
| Ln(Total Reserves) _A_ | 1.064 | 1.063 | 1.091 | 1.092 |
|  | (0.146) | (0.145) | (0.155) | (0.154) |
| Ln(Public Engagement) _A_ | 0.914 | 0.920 | 0.910 | 0.917 |
|  | (0.0688) | (0.0679) | (0.0714) | (0.0705) |
| Russell Group _A_ | 1.159 | 1.171 | 1.156 | 1.168 |
|  | (0.560) | (0.564) | (0.566) | (0.570) |
| Ln(Covid-19 Daily Cases) _A_ | 1.496** | 1.508*** | 1.367** | 1.378** |
|  | (0.238) | (0.240) | (0.214) | (0.216) |
| Days _A_ | 0.828*** | 0.827*** | 0.563*** | 0.561*** |
|  | (0.0363) | (0.0363) | (0.0374) | (0.0374) |
| Days^2^ _A_ | 1.005*** | 1.005*** | 1.011*** | 1.011*** |
|  | (0.00109) | (0.00110) | (0.00146) | (0.00146) |
| Days^3^ _A_ | 1.000*** | 1.000*** | 1.000*** | 1.000*** |
|  | (0.00000716) | (0.00000718) | (0.00000914) | (0.00000917) |
| Ln(Total Enrolment)_B_ | 0.968*** | 0.951*** | 0.992 | 0.974* |
|  | (0.0107) | (0.0115) | (0.0128) | (0.0135) |
| Proportion Income Tuition _B_ | 1.190* | 1.262*** | 2.191*** | 2.308*** |
|  | (0.114) | (0.113) | (0.268) | (0.271) |
| Ln(Total Reserves) _B_ | 1.013 | 1.019 | 0.958** | 0.966* |
|  | (0.0161) | (0.0167) | (0.0174) | (0.0190) |
| Ln(Public Interaction) _B_ | 1.010* | 1.011* | 1.025*** | 1.024*** |
|  | (0.00595) | (0.00621) | (0.00635) | (0.00682) |
| Russell Group _B_ | 0.994 | 1.031 | 1.209*** | 1.244*** |
|  | (0.0323) | (0.0292) | (0.0505) | (0.0462) |
| Ln(Covid-19 Daily Cases) _B_ | 1.410*** | 1.401*** | 1.357*** | 1.352*** |
|  | (0.0771) | (0.0765) | (0.0667) | (0.0658) |
| B Twitted _A(t-2)_ | 28.53*** | 27.55*** |  |  |
|  | (7.312) | (7.109) |  |  |
| (Neighbour far)(B Twitted _A(t-2)_) | 0.728*** |  |  |  |
|  | (0.0374) |  |  |  |
| (Neighbour close)(B Twitted _A(t-2)_) |  | 0.612*** |  |  |
|  |  | (0.0604) |  |  |
| Neighbour far |  |  | 0.769*** |  |
|  |  |  | (0.0352) |  |
| Neighbour close |  |  |  | 0.636*** |
|  |  |  |  | (0.0595) |
| Constant | 0.000115*** | 0.000130*** | 3.490 | 4.037 |
|  | (0.000110) | (0.000125) | (3.727) | (4.322) |
| Observations | 858972 | 858972 | 163670 | 163670 |
| Clusters | 141 | 141 | 140 | 140 |
| Pseudo-R2 | 0.352 | 0.352 | 0.175 | 0.175 |
| Log L | -22645.0 | -22643.2 | -20763.8 | -20754.5 |

Dependent variable: Emulation of first Covid-19 tweet. All models are discrete survival models with logit link and cubic polynomial for number of days to event. Results in odds ratios. Standard errors in parentheses clustered by university A. Oxford, Cambridge, and universities with negative total reserves are excluded from the analyses.

* *p* < 0.1, ** *p* < 0.05, *** *p* < 0.01
