# Supplementary material for: Variation in the timing of Covid-19 communication across universities in the UK
Source: PLoS One. 2021 Feb 16;16(2):e0246391. doi: 10.1371/journal.pone.0246391 (PMC7886223; doi:10.1371/journal.pone.0246391)
Supplement: S1 Appendix — (DOCX) [file pone.0246391.s008.docx]

**S1 Appendix. Specific HESA tables**

1. Student enrolments: ‘Table 1 - HE student enrolments by HE provider 2014/15 to 2018/19’ at https://www.hesa.ac.uk/data-and-analysis/students/table-1.

2. Tuition income: ‘Table 1 - Consolidated statement of comprehensive income and expenditure 2015/16 to 2018/19’ at https://www.hesa.ac.uk/data-and-analysis/students/table-1.

3. Reserves: ‘Table 3 - Consolidated balance sheet 2015/16 to 2018/19’ at https://www.hesa.ac.uk/data-and-analysis/finances/table-3.

4. Public interaction: ‘Table 5 - Social, community and cultural engagement: Designated public events by HE provider 2014/15 to 2018/19’ at https://www.hesa.ac.uk/data-and-analysis/providers/business-community/table-5.

5. Buildings: ‘Table 1 - Buildings and spaces’ at https://www.hesa.ac.uk/data-and-analysis/estates/table-1.

6. Staff: ‘Table 2 - HE staff by HE provider 2014/15 to 2018/19’ at https://www.hesa.ac.uk/data-and-analysis/staff/table-2.
